# Symptom trajectories of post-COVID sequelae in patients with acute Delta or Omicron infection in Bergen, Norway

**DOI:** 10.3389/fpubh.2024.1320059

**Published:** 2024-03-05

**Authors:** Arild Iversen, Bjørn Blomberg, Kjell Haug, Bård Kittang, Türküler Özgümüs, Rebecca Jane Cox, Nina Langeland

**Affiliations:** ^1^Chief Municipal Doctor’s Office, Bergen, Norway; ^2^Department of Clinical Science, University of Bergen, Bergen, Norway; ^3^National Centre for Tropical Infectious Diseases, Department of Medicine, Haukeland University Hospital, Bergen, Norway; ^4^Department of Global Public Health and Primary Care, University of Bergen, Bergen, Norway; ^5^Department of Medicine, Haraldsplass Deaconess Hospital, Bergen, Norway; ^6^Department of Nursing Home Medicine, Bergen Municipality, Bergen, Norway; ^7^Influenza Centre, Department of Clinical Science, University of Bergen, Bergen, Norway; ^8^Department of Microbiology, Haukeland University Hospital, Bergen, Norway

**Keywords:** COVID-19, post-COVID-19 condition, SARS-CoV-2, delta variant, omicron variant

## Abstract

**Introduction:**

A substantial proportion of the over 700 million COVID-19 cases world-wide experience long-term symptoms. The objectives of this study were to compare symptom trajectories and risk factors for post-COVID-19 condition after Delta and Omicron infection.

**Methods:**

This study consecutively recruited patients with SARS-CoV-2 infection from November 2021 to March 2022. We recorded demographics, comorbidities, vaccination status, sick leave, and 18 symptoms during acute infection and after 4 months. The primary outcome measures were symptoms during acute infection and after 4 months. Secondary outcome measures were work and school absenteeism.

**Results:**

We followed a cohort of 1,374 non-hospitalized COVID-19 patients in Bergen, Norway, at three time points. The median age was 39.8 years and 11% were children <16 years. Common acute upper respiratory symptoms waned during follow-up. Fatigue remained common from acute infection (40%) until after 4 months (37%). Four months post-infection, patients reported increased frequencies of dyspnea (from 15% during acute illness to 25% at 4 months, *p* < 0.001), cognitive symptoms (from 9 to 32%, *p* < 0.001) and depression (from 1 to 17%, *p* < 0.001). Patients infected with Omicron reported less dyspnea (22% versus 27%, *p* = 0.046) and smell/taste problems (5% versus 19%, *p* < 0.001) at 4 months follow-up than those with Delta infection. Comorbidities and female sex were risk factors for persistent dyspnea and cognitive symptoms. Ten percent reported sick leave after acute illness, and vaccination reduced the risk of absenteeism (adjusted risk ratio: 0.36, 95% confidence interval: 0.15, 0.72, *p* = 0.008).

**Conclusion:**

At 4 months, home-isolated patients infected with Omicron reported overall comparable symptom burden, but less dyspnea and smell/taste problems than Delta infected patients. Several acute symptoms waned during follow-up. It is worrying that dyspnea, neurocognitive symptoms, and particularly depression, increased significantly during the first 4 months after acute infection. Previous vaccination was protective against prolonged sick leave.

## 1 Introduction

While more than 7 million people have succumbed to the COVID-19 pandemic, the number of reported cases is over 100-fold higher (1). In this context, the prospect of a high burden of persisting symptoms in survivors is worrying, both at the individual level and from a public health perspective. Early during the pandemic, it became clear that patients hospitalized for COVID-19, especially those admitted to intensive care units (ICU) were prone to suffer long-term symptoms, coined long-COVID (2), and later named post-COVID-19 condition in the ICD10 classification (3). The post-COVID-19 condition was initially suspected to be a form of post-ICU-syndrome (4), as pre-pandemic studies indicated that half of patients treated with ventilators in an ICU setting suffered persisting physical and cognitive health problems a year after discharge (5). Subsequently, evidence surfaced that approximately half of home-isolated patients with relatively mild COVID-19 suffered persisting symptoms such as fatigue, dyspnea, and cognitive problems, for half a year (6–8). A plethora of persistent symptoms have been observed after COVID-19 (9–13). The current understanding of the post-COVID-19 condition entails the presence of new or persisting symptoms present 3 months after acute illness, and lasting for 2 months or more, and highlights fatigue, dyspnea, and cognitive symptoms as the most common presentations (3). There is evidence that COVID-19 survivors have lasting poor health outcomes at least 2 years after acute infection (7, 9, 10, 14). A separate definition has been developed for children, although with a wider range of symptoms (15).

Although much is unknown regarding the pathophysiology of post-COVID-19 condition, there is evidence for persisting neuropsychiatric symptoms (10) and lasting impact on brain structures in magnetic resonance imaging (MRI) studies (16). Elevated immune responses correspond to persisting symptoms in clinical studies (6, 7, 17), and autopsy studies allude to systemic inflammation playing a larger role than viral invasion of the central nervous system (18). We lack strong prevention strategies for long-COVID, although there is evidence for partial protective effect of leading a healthy life-style (19), vaccination (20, 21), and receiving antiviral treatment for acute COVID-19 (22).

The prevalence of post-COVID-19 condition is still uncertain due to large heterogeneity of study designs and lack of stringent case definitions. Prospective follow-up studies tend to give higher prevalence estimates (>50%) than studies of healthcare records (<15%), and hospitalized patients have higher burden of symptoms than those who have been home-isolated (12). We have previously found the presence of one or more symptoms in 55 and 46% of home-isolated patients at 6 and 12 months follow-up in case control studies (6, 7), in line with a large European meta-analysis concluding that 51% of patients in the community setting had one or more symptoms of post-COVID-19 condition at 3 months (23). However, a large Dutch study correcting for pre-existing symptoms, found a prevalence as low as 13% for persistent symptoms attributable to COVID-19 (24). There is an obvious need for further research in this field. To add to current evidence on the impact of vaccination and virus variants on post-COVID-19 condition, we performed a cohort study of COVID-patients attending outpatient services at the municipality of Bergen, Norway, comparing the Delta and Omicron waves of the pandemic.

## 2 Methods

A cohort study was performed in Bergen, the second largest city in Norway. The city has approximately 288,000 inhabitants (25). Persons attending the centralized testing facility for SARS-CoV-2 in the municipality of Bergen were invited consecutively to join the study as they tested positive for SARS-CoV-2. The recruitment lasted from 9th November 2021 to 18^th^ March 2022 covering the Delta and Omicron waves. Patients were recruited by the infection control team after voluntary, informed consent, and for children their parents or guardians consented. Those consenting were asked to complete questionnaires by way of a mobile phone. Data were collected using the SurveyXact software (Rambøll, Aarhus, Denmark), and transferred to a secure server at the University of Bergen.

The data collected included demographic variables including age and sex, information on comorbidities, medication, vaccination history, absenteeism for work or school, sick leave and symptoms. Consenting patients were asked to complete three questionnaires. The first questionnaire was completed during acute illness. Subsequently, follow-up questionnaires were distributed, asking for symptoms at 2 weeks and 4 months after symptom onset. The questionnaires asked for the presence of 18 different symptoms including fever, headache, cough, chest-pain, dyspnea, runny nose, diarrhea, abdominal pain, nausea, sore throat, taste/smell disturbance, joint and muscle pain, fatigue, difficulties with memory and concentration, anxiety and depression as previously described (6, 7). For some analyses, the term “cognitive symptom” was used for the combination of memory and/or concentration problems.

According to virological surveillance data in the municipality of Bergen, and personal communication from Dr. Kjell Haug, the Medical Officer in charge of COVID-19 surveillance in Bergen Municipality, there was a rapid transition from dominance of the Delta variant of SARS-CoV-2 to the Omicron variant from approximately 1st January 2022 (26), and debut of infection before this date was set as a proxy for infection caused by the Delta variant, and debut on or after this date for Omicron.

### 2.1 Statistical analysis

The sample size was limited by the number of consecutive COVID-19 patients who accepted to participate during the study period. The number of patients with Delta variant (*n* = 742) and Omicron variant infection (*n* = 632) who were eventually included and responded to all questionnaires gave the study a power of 96% to detect an effect size of 0.2 at a significance level of 5%. Descriptive statistics are presented. Confidence intervals and *p*-values for differences in risk of symptoms were calculated using the fmsb package in R. Risk factors for persisting symptoms were evaluated using logistic regression, adjusting for sex, age, vaccination status and the presence of comorbidities. Age was analyzed by categories (16–30, 31–45, 46–60, >60 years), to get comparably sized groups with equal age ranges in all groups (except the >60 years group), in line with our previous study (6). Age group 31–45 years as reference for logistic regression, since it was the most numerous category. All comorbidities were grouped together for logistic regression analysis. Results were presented as crude and adjusted odds ratios (OR and aOR, respectively) with 95% confidence intervals (CI). Differences in absenteeism were expressed using crude and adjusted incidence risk ratios (IRR and aIRR, respectively). Development of symptoms over time was visualized in dumbbell charts. Statistical analysis and visualization were performed in R version 4.3.0 (The R Foundation for Statistical Computing. Vienna, Austria).

The study was approved by the Regional Committee for Medical Research Ethics for Western Norway (REK 118664). The questionnaires used in the study were informed by input from patients given through several of our previous COVID-19 studies.

## 3 Results

Among 6,955 individuals asked to participate in the study, 2,411 (35%) accepted to participate and filled in the questionnaire for symptoms during the acute phase. Among the 2,411 participants who accepted to participate, 1,374 (57%) had complete follow-up as they responded to all three questionnaires at three different time-points. Distribution of age, sex, comorbidities and acute symptoms were similar among the patients followed for 4 months and the ones only reporting acute symptoms. In the following, data on patients responding at all three time points are reported. Their median age was 39.8 years (interquartile range 27.7–51.9). Women constituted 61% (*n* = 832) of the participants. Eleven percent (*n* = 148) of participants were below 16 years of age. In the total cohort, 42% (*n* = 571) of participants reported having comorbidities. The most common comorbidities were pollen allergy (17%, *n* = 239), obesity (BMI ≥ 30, 12%, *n* = 168), asthma or chronic lung disease (8%, *n* = 115), hypertension (7%, *n* = 98) and rheumatological disease (4%, *n* = 52). Overall, vaccination rates were high, with 85% (*n* = 1,170), 78% (*n* = 1,078) and 24% (*n* = 333) of study participants having received one, two or three doses of COVID vaccines, respectively. Both comorbidities and vaccination were more frequent within the adult group (Supplementary Table S1). Seven-hundred and forty-two participants (54%) had infection caused by the Delta variant of SARS-CoV-2, and 46% (*n* = 632) were infected with the Omicron variant.

**Figure 1:**
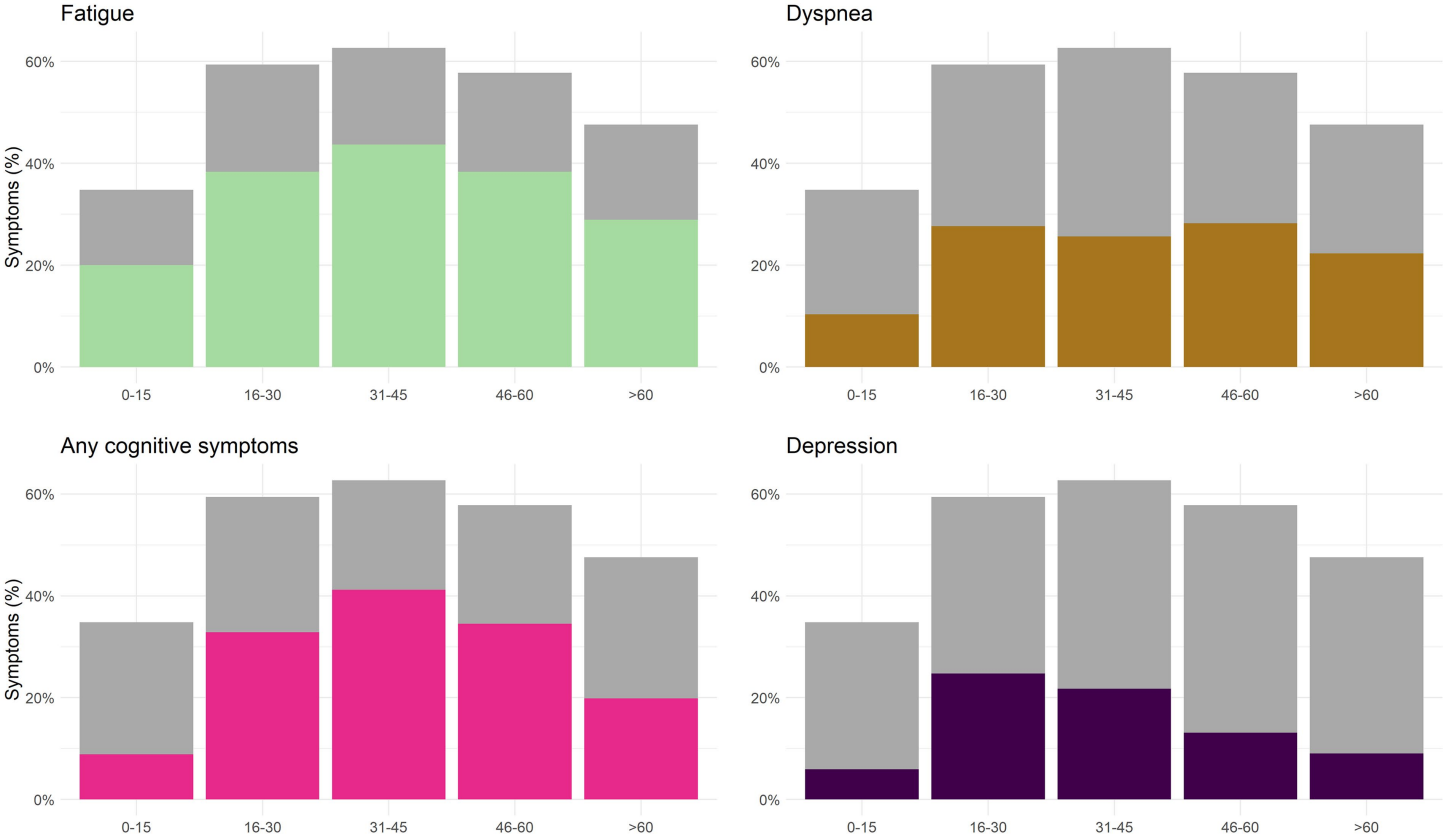
Age specific proportions of patients reporting key symptoms of post-COVID-19 condition at 4 months after acute illness. The proportion reporting any symptoms for the respective age group are shown as gray bars for comparison.

**Figure 2:**
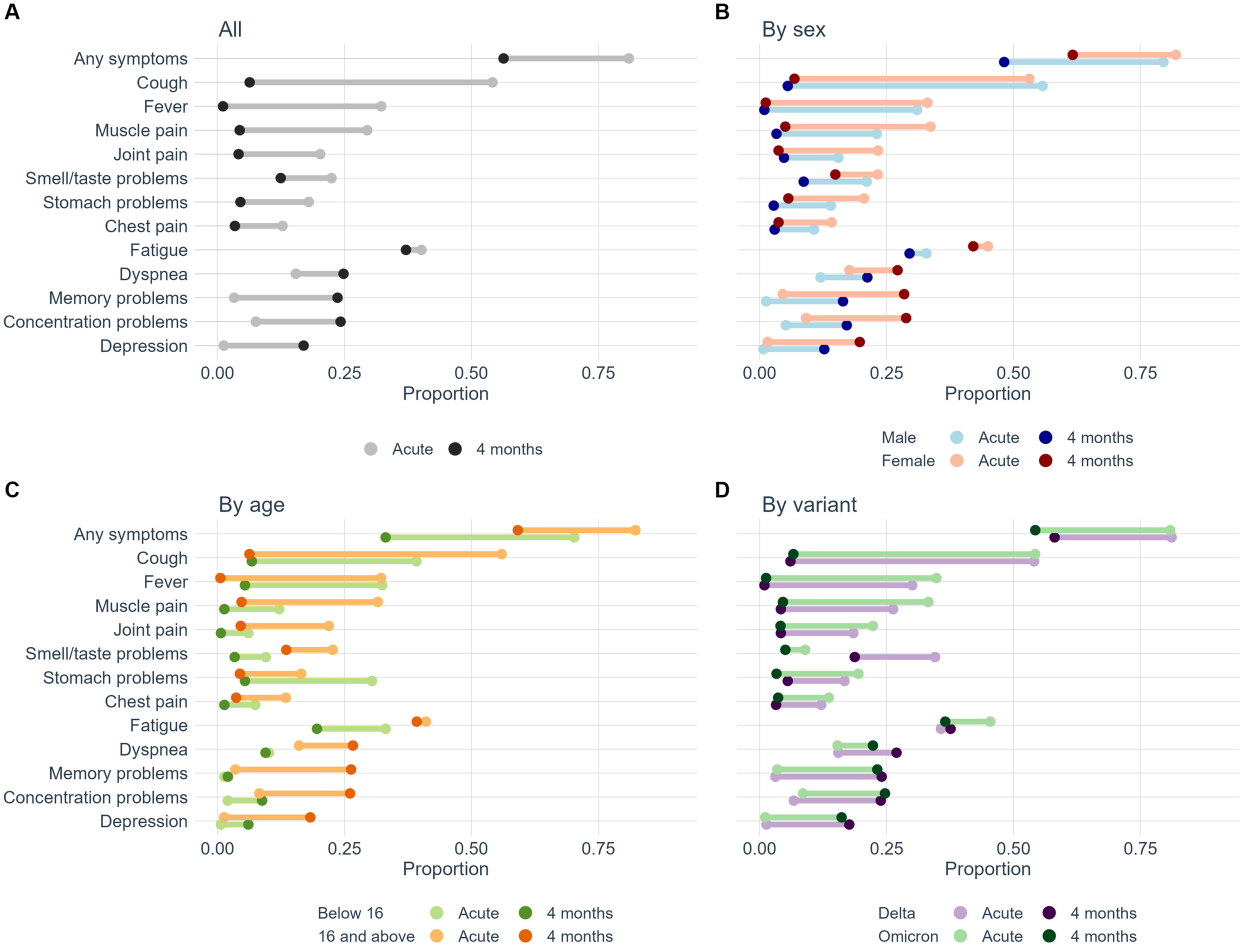
Dumbbell chart showing the trajectories of symptoms from the acute phase (light colored symbols) to 4-months follow-up (dark-colored symbols). Panel a shows change in symptoms for the whole cohort, panel b shows symptom change from acute to 4 months divided by men and women, panel c shows symptom development in children (<16 years) and adults, and panel d show symptoms for the Delta and Omicron variants.

The most common symptoms during acute infection were respiratory symptoms, such as runny nose (63%), cough (54%), headache (53%), sore throat (48%), and fatigue (40%, Table 1). At 4 months, the most common symptoms were fatigue (37%), cognitive symptoms (32%) and dyspnea (25%). Children under 16 years reported lower frequencies of acute and persisting symptoms than those who were 16 years or older (Table 2; Figure 1). At 4 months follow-up, children <16 years of age had less cognitive symptoms (26% less, *p* < 0.001), fatigue (19%, *p* < 0.001), dyspnea (18%, *p* < 0.001), depression (12%, *p* < 0.001) and smell/taste problems (11%, *p* < 0.001) than those above 16 years of age. Less than half of the participants (44%, *n* = 602) reported no symptoms at 4 months follow-up. Fatigue was frequently reported both during acute infection (40%) and after 4 months (37%). Most symptoms waned from baseline to 4 months post-infection (Table 1). However, from baseline to four-months follow-up certain key symptoms increased, including dyspnea (10% increase, *p* < 0.001), cognitive symptoms (23% increase, *p* < 0.001) and depression (16% increase, *p* < 0.001; Table 1; Figure 2).

**Table 1:** Change in key COVID-19 symptoms up to 4 months after acute infection.

	Acute *N* = 1,374	2 weeks after infection *N* = 945	4 months after infection *N* = 1,374	Change in proportions with symptoms from acute illness to 4 months
Symptom	% (*n*)	% (*n*)	% (*n*)	% (CI) *p*
Any symptoms	93% (1273)	50% (678)	56% (772)	−37% (−39, −33) *p* < 0.001
Fever	32% (443)	2% (23)	1% (15)	−31% (−34, −29) *p* < 0.001
Headache	53% (726)	22% (206)	9% (129)	−43% (−47, −40) *p* < 0.001
Cough	54% (744)	38% (355)	6% (87)	−48% (−51, −44) *p* < 0.001
Chest pain	13% (176)	12% (110)	3% (47)	−10% (−11, −7) *p* < 0.001
Dyspnea	15% (212)	22% (204)	25% (341)	**+10% (6, 12) *p* < 0.001**
Running nose	63% (860)	32% (307)	–	–
Sore throat	48% (661)	11% (107)	–	–
Smell/taste problems	22% (280)^*^	29% (275)	12% (171)	−8% (−11, −5) *p* < 0.001
Joint pain	20% (278)	6% (59)	4% (57)	−16% (−18, −13) *p* < 0.001
Muscle pain	29% (405)	9% (84)	4% (60)	−25% (−28, −22) *p* < 0.001
Fatigue	40% (552)	37% (351)	37% (510)	−3% (−7, 1) *p* = 0.100
Cognitive symptoms*	9% (118)	18% (166)	32% (440)	**+23% (21, 26) *p* < 0.001**
Memory problems	3% (45)	11% (100)	23% (316)	**+20% (17, 22) *p* < 0.001**
Concentration problems	8% (104)	14% (137)	24% (333)	**+16% (14, 19) *p* < 0.001**
Anxiety	2%(27)	2% (20)	–	–
Depression	1% (17)	2% (21)	17% (233)	**+16% (14, 18) *p* < 0.001**
Tingling	–	–	3% (40)	–
Dizziness	–	–	8% (104)	–
Sleeping problems	–	–	7% (96)	–
Palpitations	–	–	5% (67)	–
Stomach problems	18% (247)	9% (88)	5% (62)	−13% (−16, −11) *p* < 0.001

CI, 95% confidence interval. Bold font is used to mark statistically significant increase of symptoms from baseline to 4 months post-infection.*Combined memory problems and/or concentration problems.

**Table 2:** Excess risk of key symptoms in patients ≥16 years old 4 months after acute COVID-19.

	Age < 16 years *N* = 148	Age ≥ 16 years *N* = 1,226	Excess risk in patients ≥16 years
Symptom	% (*n*)	% (*n*)	% (CI) *p*
Any symptoms	34% (50)	59% (722)	25% (17, 33) *p* < 0.001
Fatigue	20% (29)	39% (481)	19% (13, 27) *p* < 0.001
Dyspnea	9% (14)	27% (327)	18% (12, 23) *p* < 0.001
Cognitive symptoms	9% (14)	35% (426)	26% (20, 31) *p* < 0.001
Depression	6% (9)	18% (224)	12% (8, 17) *p* < 0.001
Smell/taste problems	3% (5)	14% (166)	11% (7, 14) *p* < 0.001

CI, 95% confidence interval.

At 4 months follow-up, 13% (*n* = 183) of cases experienced the triad of all three key long-COVID symptoms; fatigue, dyspnea, and cognitive symptoms (difficulties with memory and/or concentration), while 49% (*n* = 673) reported one or more of these three key symptoms. While there were low rates of anxiety (2%) and depression (1%) at baseline, the proportion reporting depression at 4 months had increased by 16% (*p* < 0.001).

The overall burden of symptoms was comparable after infection with the Delta and Omicron variants (Table 3). However, at 4 months post-infection, Omicron patients reported less frequent dyspnea (22%) than patients with Delta-infection (27%, difference 5%, *p* = 0.046), and similarly less frequently disturbance of smell and taste (5% versus 19%, difference 14%, *p* < 0.001; Table 3; Figure 2). The trajectories of worsening or improvement of symptoms in patients with Delta and Omicron infection, were similar for all symptoms except for disturbance of smell and taste and, to some extent, for dyspnea and fatigue (Figure 2). Thirty-two patients (2%) reported having a new chronic disease during the first 4 months after COVID-19, irrespective of variant or prior vaccination.

**Table 3:** Difference in risk of key symptoms 4 months after acute infection Delta and Omicron variants of SARS-CoV-2.

	Delta *N* = 742	Omicron *N* = 632	Difference in risk of symptoms
Symptom	% (*n*)	% (*n*)	% (CI) *p*
Any symptoms	58% (431)	54% (341)	−4% (−9, 1) *p* = 0.124
Fatigue	38% (279)	37% (231)	−1% (−6, 4) *p* = 0.688
Dyspnea	27% (200)	22% (141)	**−5% (−9, 0) *p* = 0.046**
Cognitive symptoms	31% (232)	33% (208)	+2% (−3, 7) *p* = 0.515
Depression	18% (131)	16% (102)	−2% (−5, 2) *p* = 0.454
Smell/taste problems	19% (139)	5% (32)	**−14% (−17, −10) *p* < 0.001**

CI, 95% confidence interval. Bold font is used to mark statistically significant difference in risk of symptoms.

Risk factors for cognitive symptoms at 4 months were any comorbidity (aOR: 1.59, CI: 1.25, 2.03, *p* < 0.001), and female sex (aOR: 1.67, CI: 1.29, 2.17, *p* < 0.001; Table 4). Any comorbidity was also a significant risk factor for dyspnea at 4 months in adjusted analysis (aOR: 2.16, CI: 1.66, 2.81, *p* < 0.001). Female sex (aOR: 1.67, CI: 1.31, 2.15, *p* < 0.001) and any comorbidity (aOR: 1.53, CI: 1.21, 1.94, *p* < 0.001) were risk factors for fatigue at 4 months.

**Table 4:** Risk factors for key symptoms of post-COVID-19 condition.

Risk factors	Dyspnea		Cognitive symptoms		Fatigue	
variable	OR (CI) *p*	aOR (CI) *p*	OR (CI) *p*	aOR (CI) *p*	OR (CI) *p*	aOR (CI) *p*
Female sex	1.30 (1.00, 1.71) 0.050	1.28 (0.97, 1.69) 0.080	1.77 (1.38, 2.28) <0.001	1.67 (1.29, 2.17) <0.001	1.70 (1.33, 2.17) <0.001	1.67 (1.31, 2.15) <0.001
Age 16–30	1.19 (0.84, 1.68) 0.323	1.31 (0.92, 1.87) 0.129	0.73 (0.52, 1.00) 0.050	0.77 (0.55, 1.07) 0.120	0.84 (0.61, 1.15) 0.282	0.88 (0.64, 1.21) 0.426
31–45 (ref)	1	1	1	1	1	1
46–60	1.14 (0.83, 1.56) 0.410	1.18 (0.86, 1.63) 0.310	0.75 (0.56, 1.00) 0.047	0.78 (0.58, 1.04) 0.090	0.80 (0.60, 1.06) 0.126	0.83 (0.62, 1.11) 0.211
>60	0.83 (0.54, 1.26) 0.396	0.81 (0.52, 1.24) 0.337	0.35 (0.23, 0.53) <0.001	0.34 (0.22, 0.53) <0.001	0.52 (0.35, 0.77) 0.001	0.52 (0.35, 0.77) 0.001
Vaccination	0.98 (0.62, 1.59) 0.924	0.95 (0.59, 1.56) 0.829	1.44 (0.91, 2.33) 0.130	1.57 (0.99, 2.56) 0.064	1.54 (0.99, 2.45) 0.061	1.62 (1.04, 2.60) 0.039
Any comorbidity	2.10 (1.63, 2.73) <0.001	2.16 (1.66, 2.81) <0.001	1.55 (1.23, 1.97) <0.001	1.59 (1.25, 2.03) <0.001	1.52 (1.20, 1.92) <0.001	1.53 (1.21, 1.94) <0.001

Risk factors for post-COVID-19 condition by symptoms, unadjusted (OR) and adjusted OR (aOR) with 95% confidence intervals (CI).

As expected, the majority (*n* = 945, 77%) of subjects aged 16 years or older were absent from work during acute infection, while 120 persons (10%) were absent also after acute infection. Of these cases only 8 participants were unvaccinated with a median of 53 days absenteeism compared to 20 days in vaccinated subjects. Overall, vaccination reduced the risk of sick leave both in crude (IRR: 0.42, CI: 0.17, 0.86, *p* = 0.031) and adjusted analysis (aIRR: 0.36, CI: 0.15, 0.72, *p* = 0.008, Table 5).

**Table 5:** Risk factors for post-COVID-19 out of workdays, unadjusted (IRR) and adjusted IRR (aIRR) with 95% confidence intervals - individuals over 16.

Risk factors				
variable	IRR (CI)	*p*	aIRR (CI)	*p*
Female sex	1.09 (0.69, 1.66)	0.705	1.22 (0.79, 1.84)	0.371
Age				
16–30	0.45 (0.28, 0.77)	0.002	0.38 (0.23, 0.64)	<0.001
31–45	Ref		Ref	
46–60	0.66 (0.42, 1.04)	0.066	0.67 (0.43, 1.05)	0.082
>60	1.08 (0.52, 2.62)	0.856	1.18 (0.58, 2.80)	0.681
Vaccination	0.42 (0.17, 0.86)	0.031	0.36 (0.15, 0.72)	0.008
Omicron variant (ref: Delta)	1.09 (0.74, 1.61)	0.662	1.02 (0.71, 1.50)	0.898
Any comorbidity	0.95 (0.65, 1.41)	0.815	0.97 (0.67, 1.41)	0.887

Risk factors for post-COVID-19 sick leave in individuals over 16 years, unadjusted (IRR) and adjusted incidence risk ratios (aIRR) with 95% confidence intervals (CI).

## 4 Discussion

Several studies of post-COVID-19 condition report on symptoms after Wuhan and Alpha variants (2, 6, 8, 11–13, 27), while our study adds to the knowledgebase comparing Delta and Omicron variant infections (26, 28–32). Our data on 1,374 patients with non-severe COVID-19 shows that dyspnea and problems with smell and taste were less frequent 4 months after acute infection with Omicron variant as compared to after Delta variant infection. Other studies indicate that COVID-19 vaccination may be associated with reduced burden of post-COVID-19 condition (33). However, our finding that vaccination did not reduce the frequency of such symptoms, is also in line with other studies (34). Nevertheless, we showed that previous COVID-19 vaccination was protective against extended sick leave, which may be indirect evidence of an effect on reducing symptom burden. Neither virus variant nor comorbidity affected absence from work.

In the present study, the trajectories of symptoms fall into two groups. The most common symptoms during acute infection, such as cough, runny nose, sore throat and headache, largely wane before 4 months follow-up. On the other side, key symptoms including dyspnea, cognitive symptoms such as concentration and memory difficulties, and depression, were not common in the acute phase, but were reported more frequently at 4 months follow-up. This agrees with data in a nationwide study from Israel, where an elevated risk of dyspnea and cognitive symptoms for 12 months after acute illness was detected (35). Fatigue was equally common during the acute phase and follow-up in our material. These findings indicate that it might be useful to consider various constellations of symptoms of post-COVID-19 condition independently.

We found, in line with other studies, that female sex was a risk factors for both dyspnea and cognitive symptoms (6, 7, 23, 24, 36, 37). Possible reasons for this association include biological differences in the immune response, increased expression of angiotensin-converting enzyme-2 and transmembrane protease serine 2 receptors in women compared to men, production of pro-inflammatory interleukin-6, a greater tendency for autoimmunity in females than in males, and a potential impact of sex hormones (38, 39).

Post-COVID-19 condition is well documented in children (39, 40). We found significantly less persistent symptoms in children than in patients aged 16 years or more. While there are few studies addressing this issue, our findings support research that shows lower rates of post-COVID condition, and shorter duration of such symptoms in children (35, 40). Furthermore, a recent review identified a number of studies where old age was a risk factor for post-COVID-19 condition (37).

Similarly to other publications on sick leave after COVID-19 (41), we found a high frequency of long-term sick leave. Importantly, we found a protective effect of vaccination against extended sick leave, reducing absenteeism by over 30 days. Other authors have shown that sick-leave due to COVID-19 has much larger impact on lost workforce than sick-leave caused by side-effects of vaccines (42). Our findings suggest that, even among those who get sick with COVID-19, prior vaccination will reduce absenteeism. These findings may have implications on future vaccination strategies.

Considering each major symptom of post-COVID-19 condition alone, our results compare well to the European aggregate data for community settings for fatigue (37% in Bergen versus 31% for Europe), dyspnea (25% vs. 21%), depression (17% vs. 17%), concentration problems (24% vs. 16%), headache (9% vs. 14%), dizziness (8% vs. 10%) (23). While 13% reported having the triad of fatigue, dyspnea and cognitive symptoms, the hallmark symptoms of post-COVID-19 condition, over half of the patients reported having one or more symptoms at 4 months follow-up. A large Dutch study by Ballering et al. (24) found a somewhat lower overall frequency of any symptom at 3–5 months follow-up (41%) than in our study (56%). However, they observed a much lower frequency (13%) of symptoms deemed directly attributable to COVID-19 when controlling for pre-existing symptoms and compared to controls.

More than half of the patients experienced any one of the 18 symptoms at 4 months (56%), in line with our previous studies of the ancestral Wuhan virus (6, 7) and aggregate data from Europe (23). While we found a similar frequency of overall burden of symptoms of post-COVID-19-condition after infection with Delta and Omicron variants of SARS-CoV-2, there were less dyspnea and problems with smell and taste with Omicron infection. In a previous study of youth and adolescents we found that Omicron re-infection increased the ensuing symptom burden (26). It is challenging to decipher whether an observed reduced risk of post-COVID-19 condition after Omicron infection is due to the variant itself, or acquired natural or vaccine-induced immunity (43). However, our present data lends support to other studies documenting a trend toward lower frequency of post-COVID-19 condition after Omicron infection (28–31, 43). Our findings are in line with a Norwegian registry-based study which observed a similar symptom burden, but less dyspnea after Omicron infection (32). Differences in findings between studies may be explained by variation in the proportion of the population who have had prior COVID-19 infections, including asymptomatic infections, differences in vaccination coverage, as well as age composition and underlying comorbidity.

Our finding of increasing cognitive and depressive symptoms during the 4-month period after acute infection is of concern. While depression has been associated with post-COVID-19 condition (43, 44), studies have highlighted that post-COVID-19 depression was not associated with severity of acute illness (45), and that preexisting depression is a risk factor for developing post-COVID-19 condition (34, 46). This alludes to depression being more of a preexisting risk factor, rather than caused by COVID-19. Other studies have highlighted that depression increased in the pandemic period, regardless of exposure to SARS-CoV-2 virus (27), suggesting that societal factors, such as lock-down and other public health interventions may have played a role. A strength of our study is that, by comparing acute and long-term symptoms, we can show that depression develops after acute SARS-CoV-2 infection in as much as one in six patients. Other research has linked increased inflammation to post-COVID-19 depression (47). Similarly, studies have identified the presence of neurocognitive symptoms during acute COVID-19 as a risk factor for post-COVID condition (48). However, in our cohort, cognitive symptoms were rare during acute illness, while almost a quarter of patients developed new cognitive symptoms during the following 4 months. Worryingly, other studies have identified broader brain activation during cognitive tasks after COVID-19 (49, 50), as well as structural changes in the brain (51), and increased incidence of new-onset dementia in older adult populations after COVID-19 (52). Further research is urgently required to understand the functional and structural changes occurring in post-COVID-19 condition to define future therapeutic targets.

The lack of information on symptoms prior to infection is common in outbreak studies, and a weakness of our study. A strength of the study is, however, the consecutive inclusion of consenting non-severe COVID-19 cases at major outpatient centralized testing facilities in a geographically limited area. While we cannot rule out a potential bias in that subjects who answered at all time points may have had more long-term symptoms, our findings can help inform on the burden of post-COVID-19 condition in mild cases, and the impact of vaccination and the infecting virus variant.

## 5 Conclusion

In summary, our study found comparable overall symptom burdens after mild Delta and Omicron infection, although there was less dyspnea and problems with smell and taste after Omicron infection. Regardless of virus variants, there was a worrying increase in the number of patients reporting dyspnea, depression, and cognitive symptoms at 4 months follow-up compared to during acute infection. Other symptoms waned during the same period, and COVID-19 vaccination appeared to prevent extended sick leave.

## Data availability statement

The original contributions presented in the study are included in the article/Supplementary materials, further inquiries can be directed to the corresponding author.

## Ethics statement

The study was approved by the Regional Committee for Medical Research Ethics for Western Norway - REK 118664. The studies were conducted in accordance with the local legislation and institutional requirements. Written informed consent for participation in this study was provided by the participants’ legal guardians/next of kin.

## Author contributions

AI: Conceptualization, Data curation, Investigation, Methodology, Software, Writing – review & editing. BB: Data curation, Formal analysis, Methodology, Writing – original draft, Writing – review & editing, Visualization. KH: Conceptualization, Writing – review & editing. BK: Writing – review & editing. TÖ: Data curation, Formal analysis, Visualization, Writing – review & editing. RC: Funding acquisition, Supervision, Writing – review & editing. NL: Conceptualization, Funding acquisition, Writing – review & editing.
