# Supplementary material for: Symptom trajectories of post-COVID sequelae in patients with acute Delta or Omicron infection in Bergen, Norway
Source: Front Public Health. 2024 Mar 5;12:1320059. doi: 10.3389/fpubh.2024.1320059 (PMC10948556; doi:10.3389/fpubh.2024.1320059)
Supplement: Supplementary file 1 [file Table_1.DOCX]

Supplementary table

Characteristics of participants and the proportion reporting symptoms 4 months by age groups (years).

|  | All  N=1374 | 0-15y  N = 135 | 16-30y  N = 271 | 31-45y  N = 437 | 46-60y  N = 365 | >60y  N = 166 |
| --- | --- | --- | --- | --- | --- | --- |
| **Demographics** |  |  |  |  |  |  |
| Sex, %female | 832 (61%) | 61 (45%) | 178 (66%) | 294 (67%) | 210 (58%) | 89 (54%) |
| BMI ≥ 30^a^ | 168 (12%) | 1 (1%) | 22 (8%) | 63 (14%) | 66 (18%) | 16 (10%) |
| Any comorbidity | 571 (42%) | 25 (19%) | 93 (35%) | 193 (44%) | 169 (46%) | 91 (55%) |
| Vaccination | 1170 (85%) | 29 (21%) | 246 (91%) | 399 (91%) | 335 (92%) | 161 (97%) |
| Variant, %Delta | 742 (54%) | 85 (63%) | 129 (48%) | 233 (53%) | 214 (59%) | 81 (49%) |
| **Symptoms^b^** |  |  |  |  |  |  |
| Any symptoms | 772 (56%) | 47 (35%) | 161 (59%) | 274 (63%) | 211 (58%) | 79 (48%) |
| Triade of key symptoms^c^ | 673 (49%) | 33 (24%) | 143 (53%) | 238 (55%) | 191 (52%) | 68 (41%) |
| Fatigue | 510 (37%) | 27 (20%) | 104 (38%) | 191 (44%) | 140 (38%) | 48 (29%) |
| Dyspnea | 341 (25%) | 14 (10%) | 75 (28%) | 112 (26%) | 103 (28%) | 37 (22%) |
| Any cognitive symptom^d^ | 440 (32%) | 12 (9%) | 89 (33%) | 180 (41%) | 126 (35%) | 33 (20%) |
| Dizziness | 104 (8%) | 4 (3%) | 21 (8%) | 42 (10%) | 31 (8%) | 6 (4%) |
| Depression | 233 (17%) | 8 (6%) | 67 (25%) | 95 (22%) | 48 (13%) | 15 (9%) |
| Taste/smell disturbance | 171 (12%) | 4 (3%) | 27 (10%) | 71 (16%) | 56 (15%) | 13 (8%) |
| Headache | 129 (9%) | 14 (10%) | 30 (11%) | 43 (10%) | 29 (8%) | 13 (8%) |
| Data was collected at four months with a median (IQR) 116 (44) days after acute COVID-19  ^a^n = 1359  ^b^Symptoms with frequency more than 10% are shown in the table  ^c^any of fatigue, dyspnea, any cognitive symptom  ^d^concentration and/or memory problems | | | | | | |
